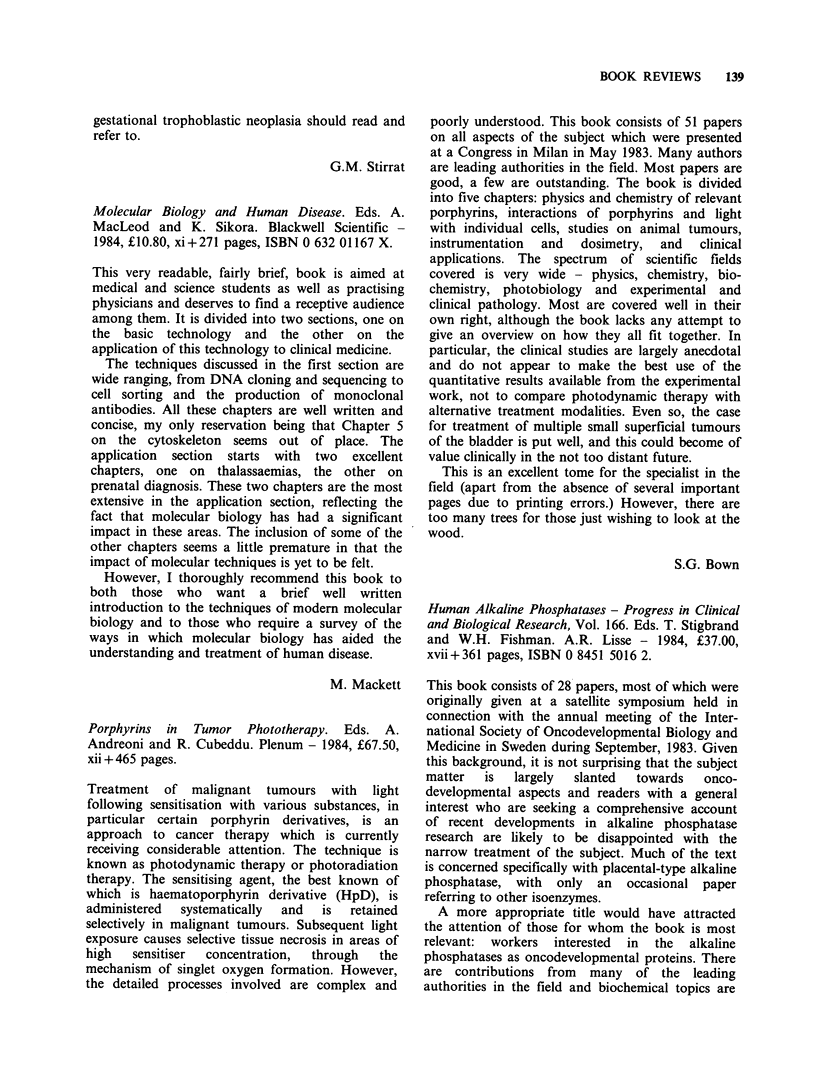# Molecular Biology and Human Disease

**Published:** 1985-07

**Authors:** M. Mackett


*Molecular Biology and Human Disease*. Eds. A. MacLeod and K. Sikora. Blackwell Scientific – 1984, £10.80, xi+271 pages, ISBN 0 632 01167 X.

This very readable, fairly brief, book is aimed at medical and science students as well as practising physicians and deserves to find a receptive audience among them. It is divided into two sections, one on the basic technology and the other on the application of this technology to clinical medicine.

The techniques discussed in the first section are wide ranging, from DNA cloning and sequencing to cell sorting and the production of monoclonal antibodies. All these chapters are well written and concise, my only reservation being that Chapter 5 on the cytoskeleton seems out of place. The application section starts with two excellent chapters, one on thalassaemias, the other on prenatal diagnosis. These two chapters are the most extensive in the application section, reflecting the fact that molecular biology has had a significant impact in these areas. The inclusion of some of the other chapters seems a little premature in that the impact of molecular techniques is yet to be felt.

However, I thoroughly recommend this book to both those who want a brief well written introduction to the techniques of modern molecular biology and to those who require a survey of the ways in which molecular biology has aided the understanding and treatment of human disease.

M. Mackett